# Supplementary material for: 3D printed graphene-based self-powered strain sensors for smart tires in autonomous vehicles
Source: Nat Commun. 2020 Oct 26;11:5392. doi: 10.1038/s41467-020-19088-y (PMC7588488; doi:10.1038/s41467-020-19088-y)
Supplement: Supplementary file 1 — Supplementary Information [file 41467_2020_19088_MOESM1_ESM.pdf]

Supplementary Information

3D Printed Graphene-based Self-Powered Strain Sensors for Smart Tires in Autonomous Vehicles

Deepam Maurya[1,2*], Seyedmeysam Khaleghian[3], Rammohan Sriramdas[4], Prashant Kumar[1], Ravi Anant Kishore[1,5], Min Gyu Kang[4], Vireshwar Kumar[6,7], Hyun-Cheol Song[8], Seul-Yi Lee[9], Yongke Yan[4], Jung-Min Park[6], Saied Taheri[1,10*], and Shashank Priya[4*]

[1]Department of Mechanical Engineering, Virginia Tech, Blacksburg, VA 24061, United States.

[2]Department of Materials Science and Engineering, Virginia Tech, Blacksburg, VA 24061, United States.

[3]Department of Engineering Technology, Texas State University, San Marcos, TX 78666, United States

[4] Department of Materials Science and Engineering, Penn State University, University Park, PA 16802, USA

[5]National Renewable Energy Laboratory, 15013 Denver West Pkwy, Golden, CO 80401, USA.

[6]Department of Electrical and Computer Engineering, Virginia Tech, Blacksburg, VA 24061, USA

[7]Department of Computer Science and Engineering, Indian Institute of Technology Delhi, New Delhi, 110016, India

[8]Center for Electronic Materials, Korea Institute of Science and Technology (KIST), Seoul 02792, Republic of Korea

[9]Institute for Critical Technology and Applied Science (ICTAS), Virginia Tech, Blacksburg, Virginia 24061, United States.

[10]Center for Tire Research (CenTiRe), Virginia Tech, Blacksburg, VA 24061, United States.

*Corresponding authors: mauryad@vt.edu, staheri@vt.edu, sup103@psu.edu

Supplementary note 1. 3D printing process optimization

The process included optimization of the number of passes along with the printing speed. The gas flow in the atomizer and sheath pressure was adjusted to tune the printing resolution. We noticed that on Kapton film, three passes were enough to achieve homogeneous film and consistent electrical property. In order to check the functionality, first, we deformed the printed sensors with hand and measured the change in resistance with stretching. Interestingly, the results indicated an almost linear change in resistance with stretching (Supplementary Fig. 2). Next, we attached Kapton with a printed sensor on an aluminum beam for measuring the strain and corresponding change in resistance. The aluminum beam was fixed at one end near the printed sensor, while another end was used for suspending the weight in order to generate strain (Supplementary Fig. 3). The change in resistance was plotted as a function of strain (Supplementary Fig. 4). This change was linear with gauge factor close to that of other metal-based strain sensors. The strain was calculated using expression:

$$\epsilon = \frac{6}{E}\frac{\left(\frac{33}{140}\rho bhl + m_{\mathrm{tip}}\right)g(l-x)}{bh^2} \quad (1).$$

The strain gauge factor was calculated using expression:

$$\mathrm{GF} = \frac{\frac{\Delta R}{R}}{\epsilon} \quad (2).$$

Various parameters in these expressions are described in Supplementary Fig.3*c*. The calculated value of the gauge factor was found to be 1.49, which is same as that of the metal-based strain gauges. However, these metal nanoparticle-based sensors have challenges in withstanding the deformation of a tire and thus other materials are needed to provide required robustness.

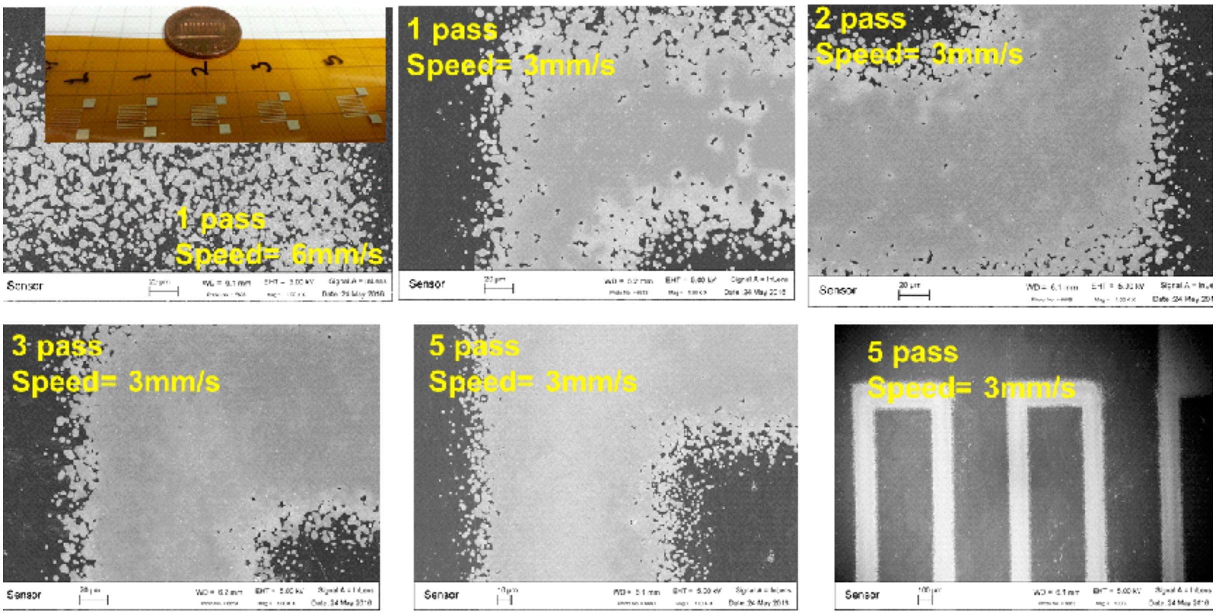

Supplementary Fig. 1 3D prining process optimization. SEM micrographs of the 3D printed strain sensors using silver naoparticle based ink with different number of passes.

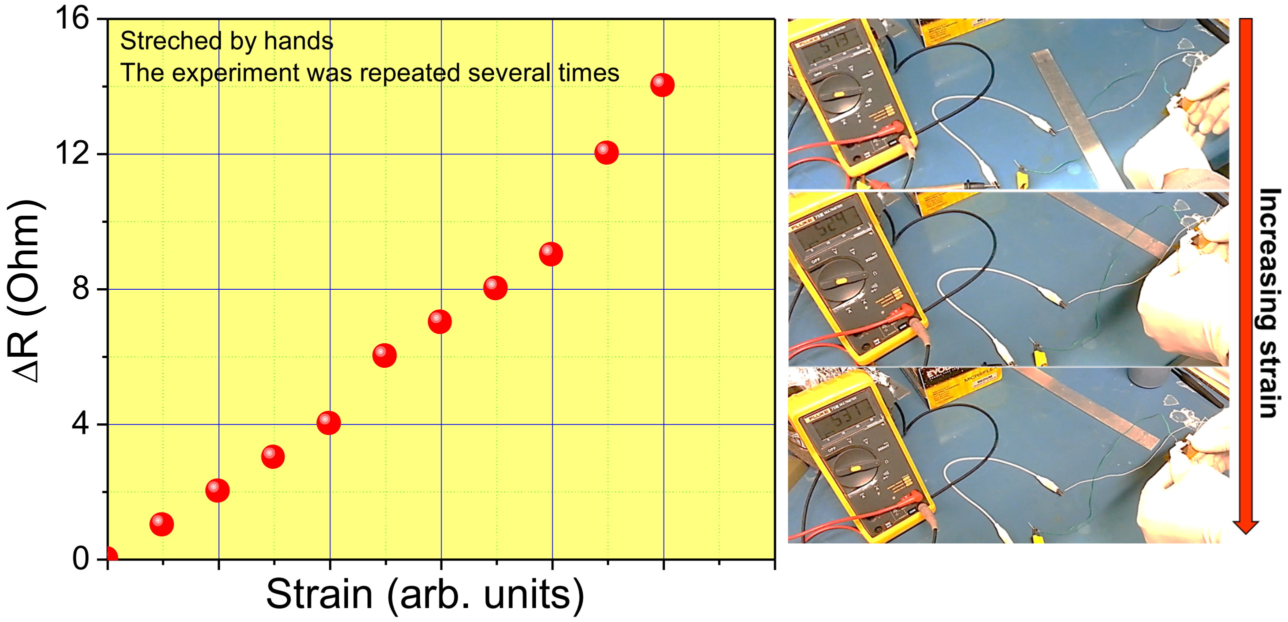

Supplementary Fig. 2. Results on 3D printed sensor using silver nano particles. Change is resistance with respect to stretching using hand.

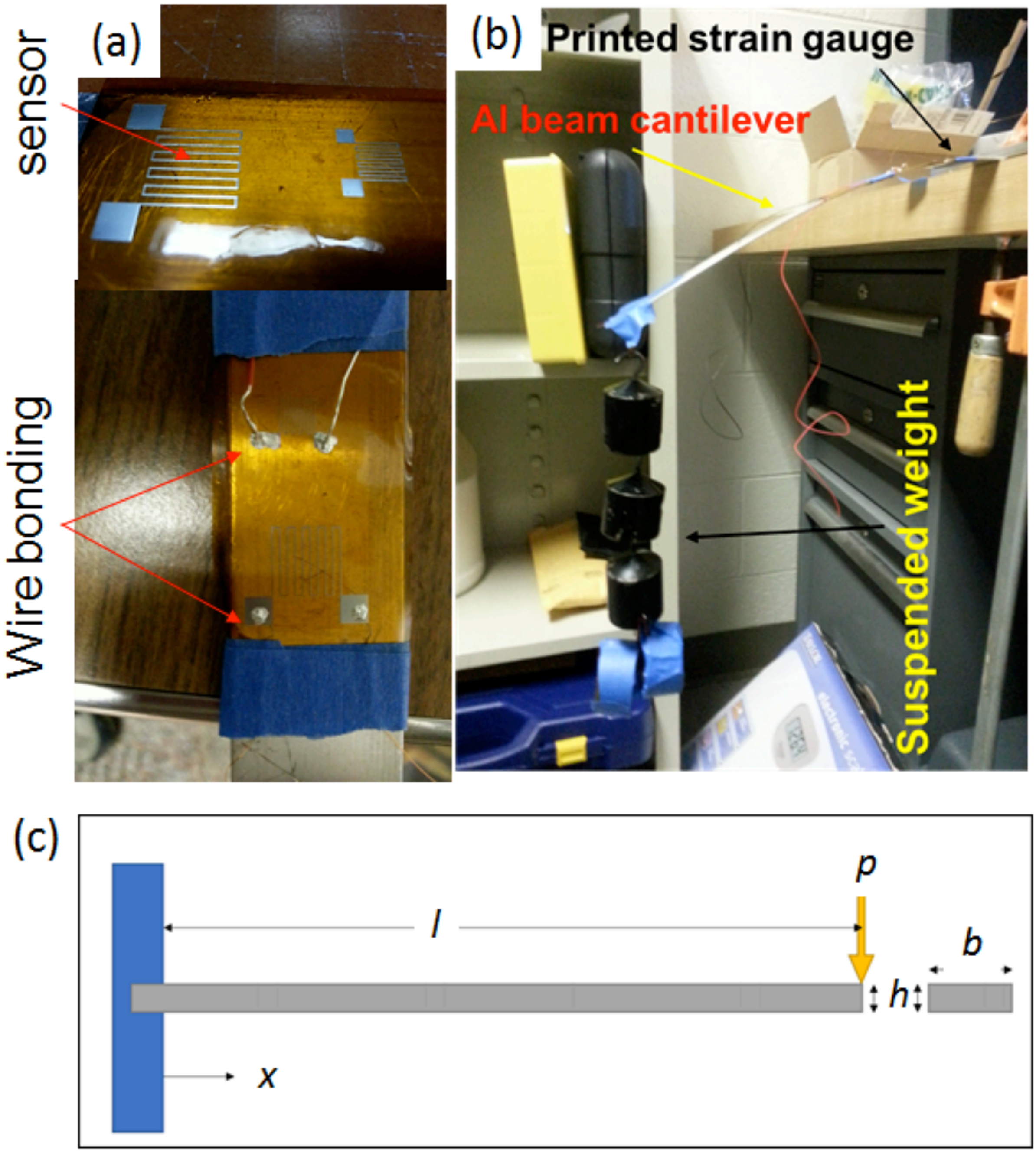

Supplementary Fig. 3 Strain measurement using printed sensor. **a** Silver-based strain sensors printed on Kapton attached to an Aluminum beam. **b** Photograph of the experimental setup for creating strain in a beam by suspending weights, and measuring corresponding resistance change. **c** Schematic of the beam marked with different geometrical values for calculating strain.

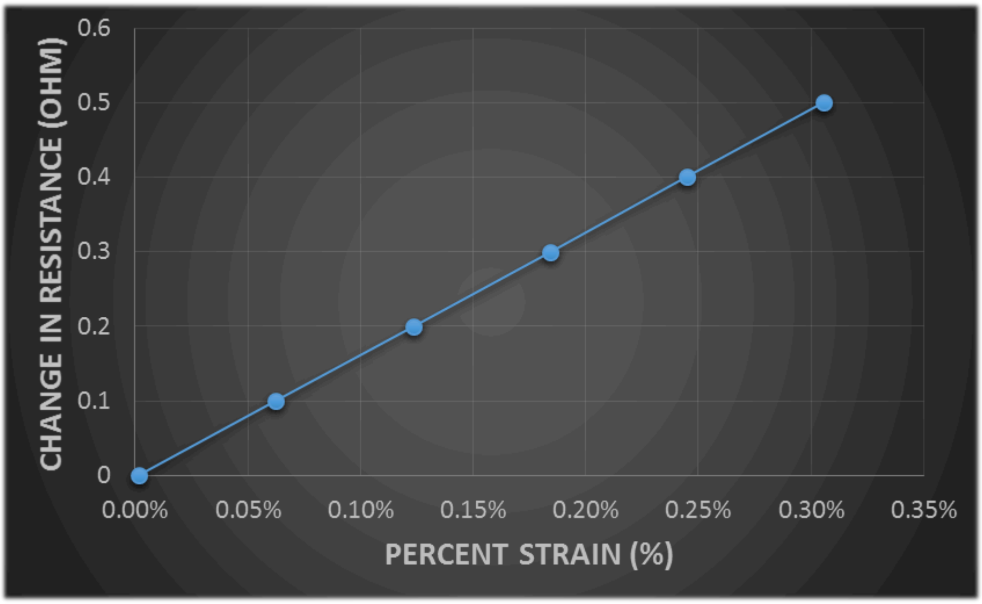

Supplementary Fig. 4. Performance of silver-based 3D printed sensor. Resistance change versus strain plot for the silver based piezoresistive sensor measured using configuration shown in Supplementary Fig. 3*b*. Please note linear change in resistance for the given values of strain.

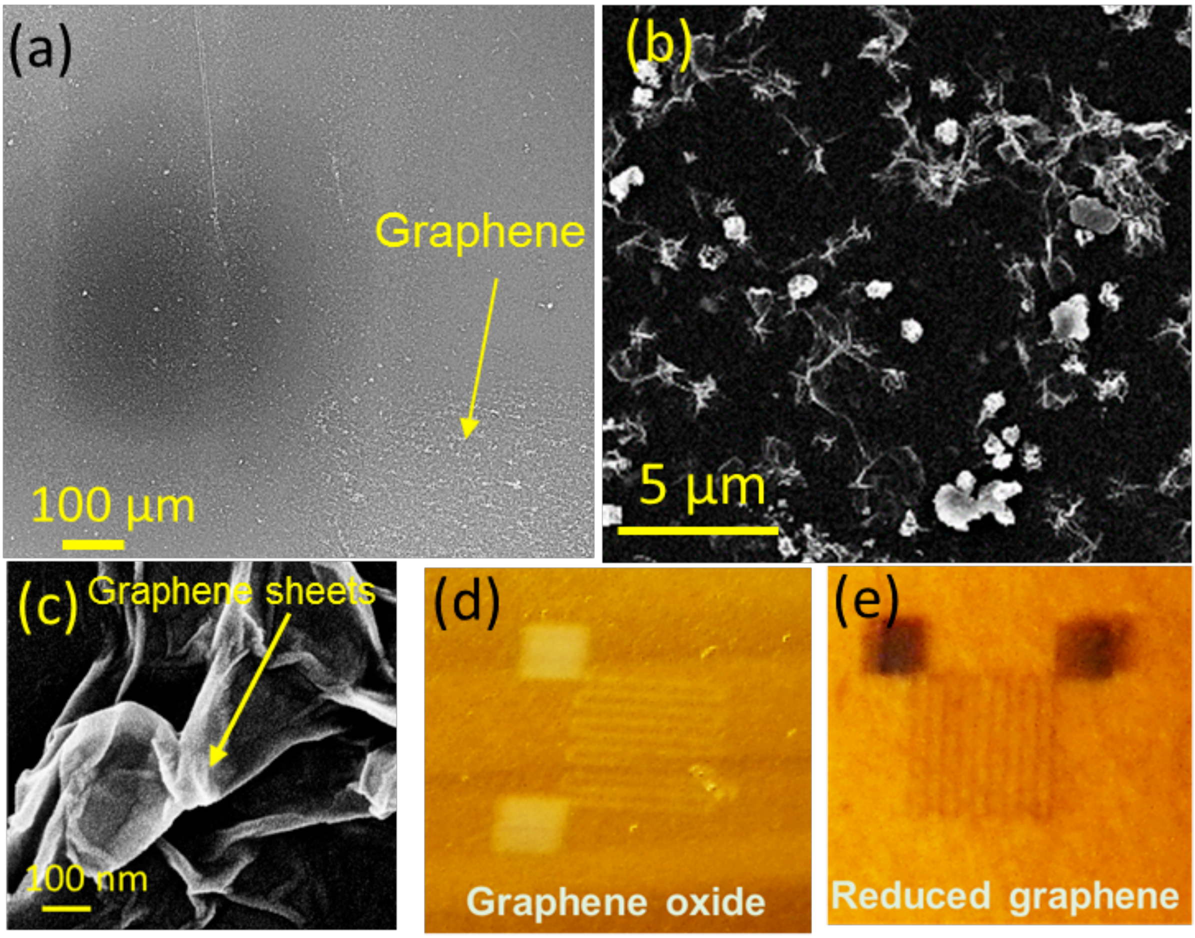

Supplementary Fig. 5 Morphology of the printed graphene based sensor. **a-c** SEM micrographs of the printed GO based sensor at different magnifications. The microstructure is not homogeneous due to unoptimized process. Optical image of the printed sensor architectures for **d** GO and **e** rGO.

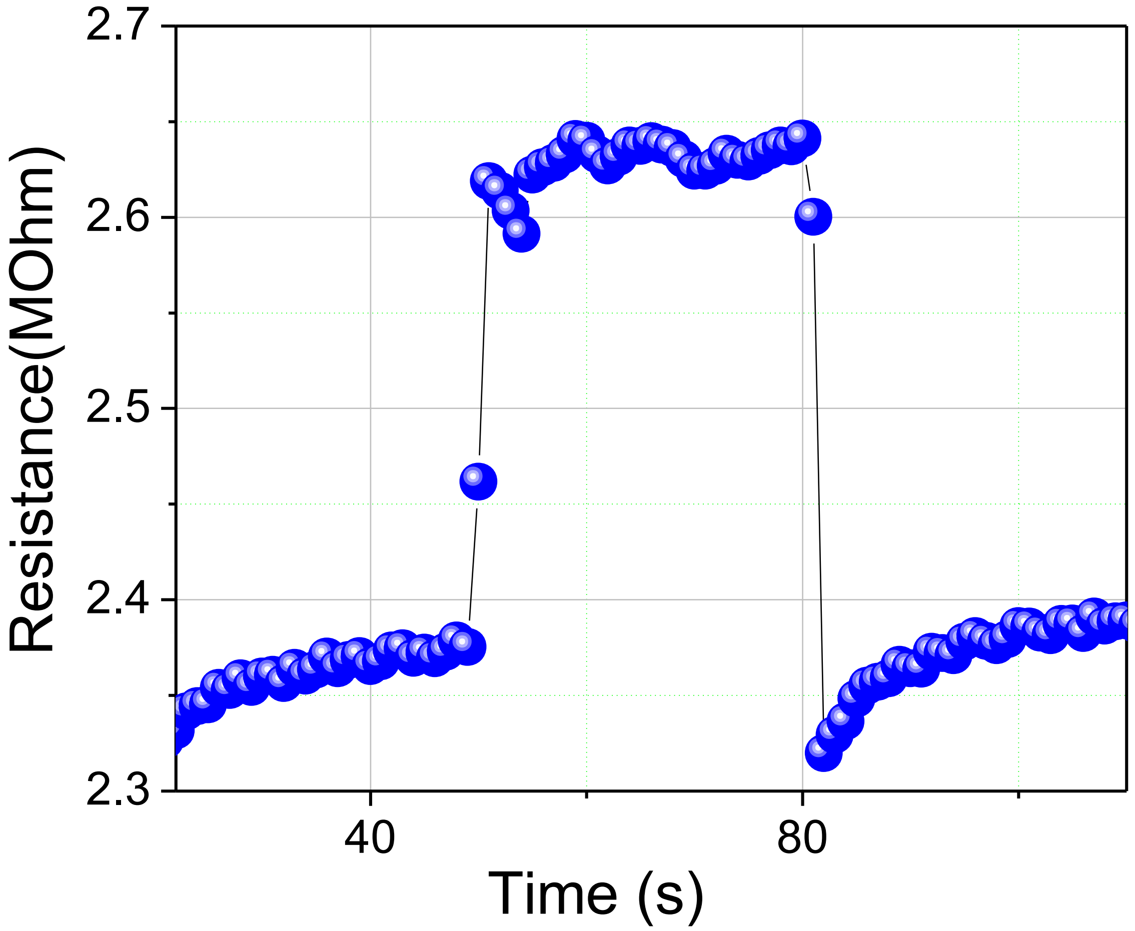

Supplementary Fig. 6 Resistance change for rGO sensor. Time dependent change in the resistance of the 3D printed rGO based sensor under tensile strain.

Supplementary Table 1- Summary of the experiments performed in real life environment during field test.

Tire pressure (psi)	Normal load (N)	Speed (MPH)
20	2000	5, 10 and 15
	3000	5, 10 and 15
	4000	5, 10 and 15
25	2000	5, 10 and 15
	3000	5, 10 and 15
	4000	5, 10 and 15
28	2000	5, 10 and 15
	3000	5, 10 and 15
	4000	5, 10 and 15

Supplementary Table 2. Calculation of no. of cycles of 3D printed sensor during field test.

	5 mph	Time difference	10 mph	Time difference	20 mph	Time difference	
**2000 N 28 PSI**	12:15:22 PM		12:55:12 PM		12:57:25 PM		
	12:51:47 PM	0:36:25	12:56:27 PM	0:01:15	12:59:10 PM	0:01:45	
**3000 N 28 PSI**	1:01:51 PM		1:10:35 PM		1:16:29 PM		
	1:03:27 PM	0:01:36	1:12:40 PM	0:02:05	1:17:51 PM	0:01:22	
**4000 N 28 PSI**	1:19:12 PM		1:22:21 PM		1:24:36 PM		
	1:21:38 PM	0:02:25	1:24:13 PM	0:01:52	1:25:57 PM	0:01:21	
**2000 N 25 PSI**	1:32:45 PM		1:36:49 PM		1:39:58 PM		
	1:35:36 PM	0:02:52	1:38:43 PM	0:01:54	1:41:02 PM	0:01:04	
**3000 N 25 PSI**	1:44:01 PM		1:58:40 PM		2:05:53 PM		
	1:58:14 PM	0:14:13	2:04:40 PM	0:06:00	2:06:52 PM	0:00:59	
**4000 N 25 PSI**	2:08:37 PM		2:14:57 PM		2:16:57 PM		
	2:13:44 PM	0:05:06	2:15:58 PM	0:01:01	2:18:11 PM	0:01:14	
**2000 N 20 PSI**	2:23:48 PM		2:27:09 PM		2:29:25 PM		
	2:25:49 PM	0:02:02	2:28:16 PM	0:01:07	2:30:33 PM	0:01:09	
**3000 N 20 PSI**	2:32:09 PM		2:40:59 PM		2:43:29 PM		
	2:33:59 PM	0:01:50	2:42:09 PM	0:01:10	2:44:29 PM	0:01:01	
**4000 N 20 PSI**	2:46:18 PM		2:48:58 PM		2:51:19 PM		
	2:47:23 PM	0:01:05	2:50:04 PM	0:01:05	2:52:14 PM	0:00:54	
**Total running time**		1:07:34		0:17:29		0:10:48	
**Wheel perimeter (Inch)**		96		96		96	
**Running distance (miles)**		5.63		2.91		3.6	Total no. of cycles =
**No of cycles**		3723		1927		2380	8031

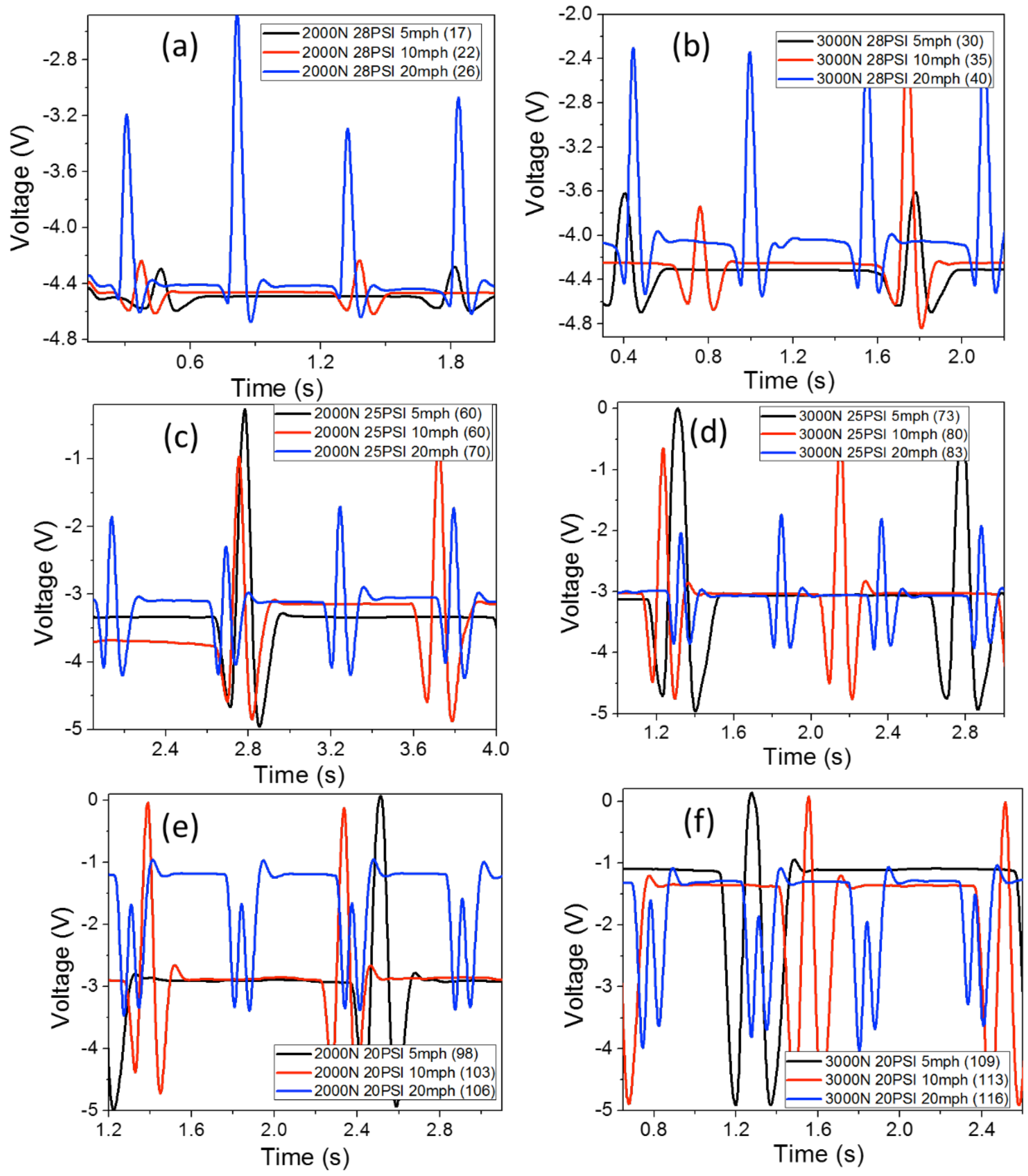

Supplementary Fig. 7 Field test results of the 3D printed graphene based sensor. Voltage output across a 3D printed piezoresistive sensor at three different speeds and two different normal loads at (a)-(b) 28 psi, (c)-(d) 25 psi, (e)-(f) 20 psi.

Supplementary note 2. Simulation and experimental result matching. As shown in Supplementary Fig. 3*c*, in each rotaion, tire has compressive strain (zone P or Q) and tensile strain (zone R). For comparing experimental and modeling results, we define the ratio (K) as:

$$K = \frac{Compressive\ zone\ amplitude\ (C)}{Tesnile\ zone\ amplitude\ (T)}$$

We calculated the experimental K value and compared this ratio with our modeling results. Supplementary Fig. 8 shows the experimental and modeling results on the same x-axis. We found that $K_{\mathrm{Experiment}}$ = 0.25 and K_{Model} = 0.174. This matching is quite significant considering that the data was collected in real enviornment in contrast to controlled lab enviorment.

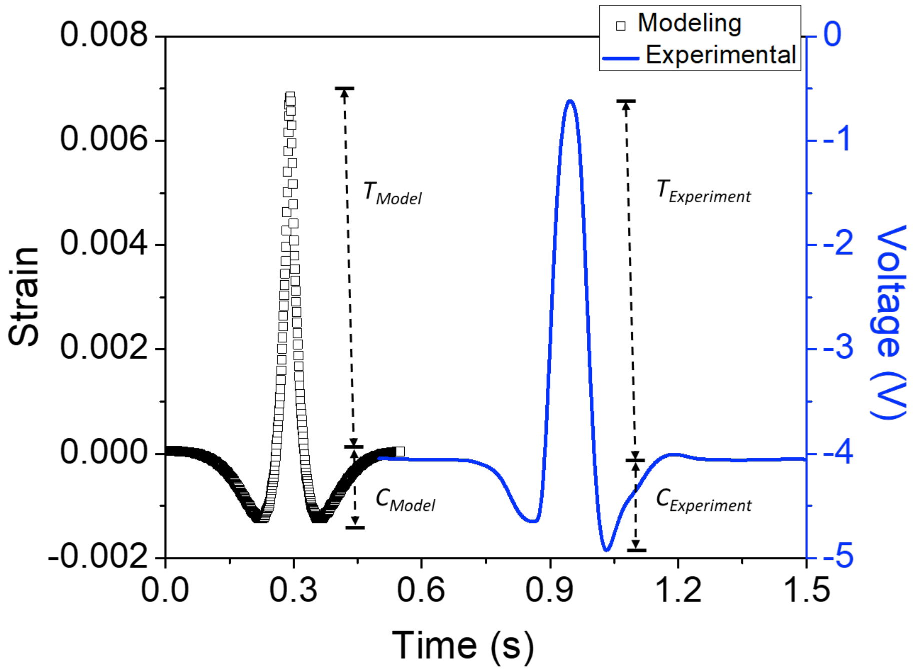

Supplementary Fig. 8 Simulation and experimental results. Simulation results for the strain and experimental results (in voltage) when the tire pressure is 28 psi for a 4000 N load at 5 mph. Updown arrows show the tensile and compressive strain amplitude of modeling and experimental results.

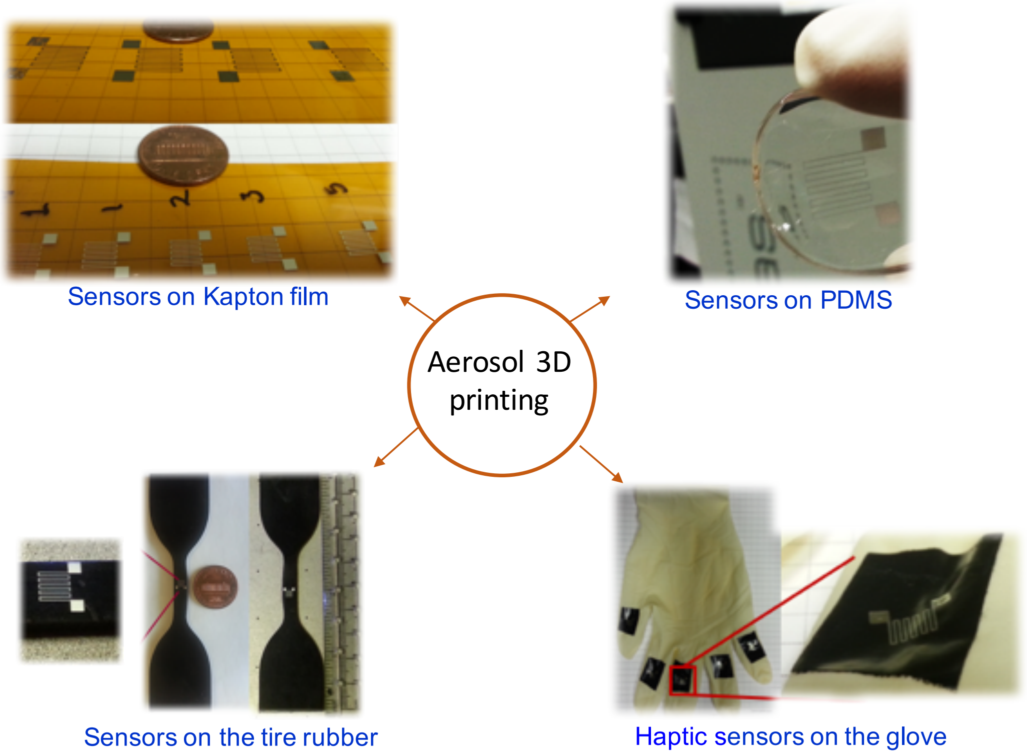

Supplementary Fig. 9 Printed sensors on various substrates. 3D printed sensors on various substrates using aerosol deposition process.

Supplementary note 3. **Cost estimation of the graphene based 3d printed sensor**. In order to estimate the cost of a 3D printed graphene based sensor, material cost and electricity were considred as leading factors. The cost of one such printed sensor was estimated to be around 2.7 cents. The scaling of the processing will further reduce the cost of the sensor due to lower raw material cost.
