# Supplementary material for: 3D printed graphene-based self-powered strain sensors for smart tires in autonomous vehicles
Source: Nat Commun. 2020 Oct 26;11:5392. doi: 10.1038/s41467-020-19088-y (PMC7588488; doi:10.1038/s41467-020-19088-y)
Supplement: Supplementary file 3 — Description of Additional Supplementary Files [file 41467_2020_19088_MOESM3_ESM.pdf]

Description of Additional Supplementary Files

File name: Supplementary Movie 1
Description: Field test experimental setup for collecting data from 3D printed strain sensor in real life environment

File name: Supplementary Movie 2
Description: Demonstration of wireless data transfer powered by energy stored from the piezoelectric tire energy harvester
